# Multimorbidity and long-term disability and physical functioning decline in middle-aged and older Americans: an observational study

**DOI:** 10.1186/s12877-022-03548-9

**Published:** 2022-11-28

**Authors:** Carole E. Aubert, Mohammed Kabeto, Navasuja Kumar, Melissa Y. Wei

**Affiliations:** 1 Department of General Internal Medicine, Inselspital, Bern University Hospital, University of Bern, Bern, Switzerland; 2 Institute of Primary Health Care (BIHAM), University of Bern, Bern, Switzerland; 3 Department of Internal Medicine, University of Michigan, Ann Arbor, MI USA; 4 Division of General Internal Medicine and Health Services Research, Department of Medicine, David Geffen School of Medicine, University of California, Los Angeles, CA USA; 5 Greater Los Angeles Veterans Healthcare System, CSHIIP, Los Angeles, CA USA

**Keywords:** Multiple chronic conditions, Autonomy, Functional independence, Activities of daily living, Functional decline

## Abstract

**Background:**

Multimorbidity is highly prevalent and associated with several adverse health outcomes, including functional limitations. While maintaining physical functioning is relevant for all adults, identifying those with multimorbidity at risk for faster rates of physical functioning decline may help to target interventions to delay the onset and progression of disability. We quantified the association of multimorbidity with rates of long-term disability and objective physical functioning decline.

**Methods:**

In the Health and Retirement Study, we computed the Multimorbidity-Weighted Index (MWI) by assigning previously validated weights (based on physical functioning) to each chronic condition. We used an adjusted negative binomial regression to assess the association of MWI with disability (measured by basic and instrumental activities of daily living [ADLs, IADLs]) over 16 years, and linear mixed effects models to assess the association of MWI with gait speed and grip strength over 8 years.

**Results:**

Among 16,616 participants (mean age 67.3, SD 9.7 years; 57.8% women), each additional MWI point was associated with a 10% increase in incidence rate of disability (IRR: 1.10; 95%CI: 1.09, 1.10). In 2,748 participants with data on gait speed and grip strength, each additional MWI point was associated with a decline in gait speed of 0.004 m/s (95%CI: -0.006, -0.001). The association with grip strength was not statistically significant (-0.01 kg, 95%CI: -0.73, 0.04). The rate of decline increased with time for all outcomes, with a significant interaction between time and MWI for disability progression only.

**Conclusion:**

Multimorbidity, as weighted on physical functioning, was associated with long-term disability, including faster rates of disability progression, and decline in gait speed. Given the importance of maintaining physical functioning and preserving functional independence, MWI is a readily available tool that can help identify adults to target early on for interventions.

**Supplementary Information:**

The online version contains supplementary material available at 10.1186/s12877-022-03548-9.

## Introduction

For older adults, maintaining physical functioning is fundamental to preserve functional independence and delay the adverse consequences of physical functioning decline, such as increased risk of hospitalization, institutionalization, and premature mortality [1, 2]. Prior studies report that objective measures of worse physical functioning are associated with several adverse short and long-term outcomes. For example, decreased gait speed was associated with increased risk of disability, recurrent falls, hip fracture, nursing home admission in older adults, and mortality [3–5]. Similarly, decreased upper body strength assessed through grip strength was associated with functional limitations and disability in older adults, poor health-related quality of life, and increased mortality risk [6–8]. Avoiding disability is universally valued, and recent analyses have highlighted disability-free survival as an important health outcome over mere overall survival [9, 10].

Physical functioning may reflect the cumulative, integrative impact of chronic conditions on health, given strong and persistent associations between multimorbidity (and its severity) and physical functioning. For example, multimorbidity is strongly associated with physical functioning decline, frailty, worse health-related quality of life, and mortality [11–15]. Further, patients themselves identify poor physical functioning as a problem related to having multiple chronic conditions [16]. Given the increasing prevalence of multimorbidity, [17–20] there is a critical need to identify individuals at risk for faster rates of long-term physical functioning decline and disability.

While previous studies reported an association between multimorbidity and functional impairment, they include several limitations such as the assessment of multimorbidity through a simple count of chronic conditions, no or short follow-up, lack of analysis of rates of decline as multimorbidity evolves over time, and lack of assessment of physical functioning through both subjective and objective measures [11].

We previously developed and validated the Multimorbidity-Weighted Index (MWI), a measure that weights chronic conditions by their impact on physical functioning [21, 22] The MWI was associated with mortality, long-term physical functioning and cognitive functioning decline, higher risk of suicide, mortality, reduced physical and mental health-related quality of life, and cross-sectionally with disability (as measured by basic Activities of Daily Living [ADLs] and Instrumental ADLs [IADLs] limitations) [21, 23–25] The MWI has been extensively compared with other measures of multimorbidity (e.g., disease count, Charlson Comorbidity Index and Elixhauser Comorbidity Index) [21, 24, 26–28].

In this study, we sought to better characterize the association of multimorbidity with rates of long-term disability and objective measures of physical functioning in a large sample representative of the US population. We used a weighted measure of multimorbidity and assessed its evolution over time. We included both subjective and objective measures of physical functioning.

## Methods

### Study population

We used data from the Health and Retirement Study (HRS), an ongoing prospective cohort of more than 38,000 US adults aged 50 years and older at enrolment, and followed up since 1992 [29]. The HRS uses geographical stratification, clustering, and oversampling of Black, Hispanic, and minority households, and is representative of about 95 million US adults. Biennially, participants answer questions about income, employment, marital and insurance status, health status and behaviors (e.g., smoking status and physical activity), physician-diagnosed medical conditions, and disability (as measured by ADLs and IADLs). In addition, since 2006, participants receive in-person objective assessments of physical functioning (including grip strength and gait speed) every four years (half random samples alternate every other wave so all participants receive repeated measures longitudinally) [30].

For the present analysis, we used all HRS participants interviewed in the year 2000 wave, aged 52 or older, with complete information on chronic diseases to compute the MWI and outcome data, and information available for at least one of the biennial follow-up waves conducted until 2016. For the disability outcome analysis, we included participants interviewed in the year 2000, with follow-up waves until 2016. For gait speed and grip strength, we included HRS participants interviewed in the year 2006, with follow-up until 2014.

This study was approved by the University of Michigan Institutional Review Board (HUM00128383) and the University of California at Los Angeles Institutional Review Board (IRB#21–001,806).

### Multimorbidity measurement and assessment

Our main exposure of interest was multimorbidity weighted on physical functioning, measured using the MWI, which was described in detail previously [22]. Briefly, we developed and validated the MWI by attributing a weight to 74 chronic conditions, according to their cross-sectional associations with physical functioning assessed with the Short Form-36 Health Survey (SF-36) [31]. A one-point increase in the MWI conveniently calibrated to approximately a one-point decrease in the SF-36 physical functioning scale, where a 2–3 point decline may be considered clinically meaningful [24].

In the HRS, participants were asked at each study wave whether a doctor had ever told them that they had the following conditions (binary answer for each condition): myocardial infarction, angina, congestive heart failure, arrhythmia, other heart problems, stroke, hypertension, chronic lung disease, cancer (excluding skin), diabetes, arthritis, hip replacement, knee replacement, dementia, and glaucoma. The information obtained for these 15 conditions at each study wave was used to compute the MWI (according to Wei et al., Supplemental S1) [21] at each wave for each participant. The MWI was calculated in a cumulative manner that carried forward chronic conditions. In other words, once a participant was diagnosed with any of the 15 conditions, he or she was considered to have the condition in subsequent waves. If a participant had missing information for any of the 15 conditions, then the data from previous visits was used to impute missing values.

### Covariate measurement and assessment

The following covariates were used in the models: age (HRS interview date *minus* birth date), sex (male, female), ethnicity (Non-Hispanic White, Non-Hispanic Black, Hispanic, other ethnicity – including Alaska Native, Asian, Native Hawaiian, Pacific Islander and any other ethnicity), education (less than high school, high school, college, ≥ 4 years of college), body mass index (BMI; < 18.5 kg/m^2^, 18.5–24.9 kg/m^2^, 25–29.9 kg/m^2^, ≥ 30 kg/m^2^), living arrangement (married, unmarried living with another person, unmarried living alone), net worth in quartiles, smoking status (never smoked, past smoker, current smoker), and follow-up time (years since baseline). BMI and smoking had missing observations. For individuals with missing BMI for a visit, BMI from the previous visit was used. If previous visit BMI was also missing, then the average BMI of all visits was used for imputation. For individuals with missing smoking data, the information from previous visit was used. Covariate information from baseline were used in the models.

### Outcomes

Our outcomes included subjective assessment of disability and two objective measures of physical functioning.

### Assessment of subjective measure of physical functioning: disability

Disability is commonly defined as difficulty with performing tasks required for independent living such as ADLs and IADLs [32]. In the HRS, self-reported limitations in ADLs included bathing, ambulating, eating, dressing, transferring, and toileting. Self-reported limitations in IADLs included meal preparation, telephone use, taking medications correctly, managing finances, and transportation. Our measure of disability was the sum of the total number of limitations in ADLs (range 0–6) plus IADLs (range 0–5), for a total range of 0–11, where 0 denotes no limitations and 11 denotes limitations with all ADLs and IADLs.

### Assessment of objective measures of physical functioning

Objective measures of physical functioning included grip strength and gait speed, which were measured every other wave since 2006 in a sample of HRS participants. Grip strength was assessed in all participants of the sample, except if they reported having had surgery, swelling, inflammation, severe pain or injury in both hands in the past six months [33]. It was measured with a Smedley spring-type hand dynamometer and defined as the best of four measurements (two in each hand). Results were presented in kilograms. Gait speed (or timed walk test) was assessed in all participants of the sample aged 65 years or older without any problems from recent surgery, injury, or other health conditions that might prevent them from walking [33]. Gait speed was measured during a 2.5-m (98.5-inch) walk test, and defined as the highest speed (in meters per second) of two attempts.

### Statistical analyses

The dependent and independent continuous variables were examined for normality and outliers. The number of ADLs and IADLs limitations was skewed (most participants had no limitations), and transforming did not improve the distribution. We therefore used mixed-effects negative binomial regression with random intercept and slope to assess the association between time-varying MWI and disability [34]. This model considers the outcome as a count variable and adjusts for overdispersion. We presented the ADL and IADL results as incidence rate ratios (IRRs) with 95% confidence intervals (CIs). Using MWI as time-varying (i.e., accounting for MWI measure at each wave) allowed us to assess whether the progression of disability and decline in physical functioning is associated with the progression in multimorbidity.

We used a linear mixed-effects model with random intercept to assess the association between time-varying MWI and changes in gait speed and grip strength, respectively. Five observations had very high values of gait speed. These were considered as outliers and excluded from the analysis. The sample size did not change when these outliers were excluded (because of repeated measures). We presented the results as regression coefficient with 95% CI.

MWI and all outcomes were modelled as time-varying variables. We conducted two different models for each of the three outcomes. Model 1 was adjusted for all covariates, as well as the outcome at baseline (i.e., baseline disability, baseline gait speed, or baseline grip strength, respectively). Baseline covariates were not modeled as time-varying.

**Fig. 1:**
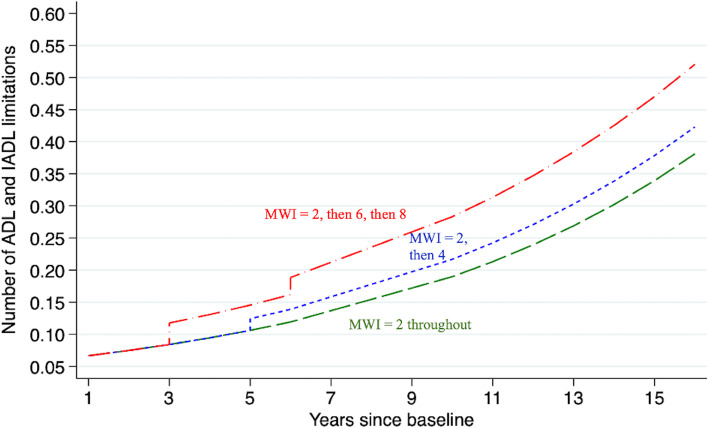
Predicted disability for exemplary HRS participants with different MWI trajectories. Abbreviations: MWI, Multimorbidity-Weighted Index. Legend: This figure illustrates the long-term progression of disability over time according to the evolution of multimorbidity. Participant-specific predicted values of disability are based on negative binomial regression with random intercept and time-varying MWI as the predictor. Models were adjusted for all baseline covariates, and baseline disability was set at the mean. Green line indicates the association between MWI and disability for a constant MWI over the whole time period, i.e., without an increase in MWI over time or interaction between MWI and time. Blue line indicates the association between MWI and disability for an increasing MWI at one time point, as if there were no interaction between MWI and time (as compared to the red line). Red line indicates the association between MWI and disability for an increasing MWI at several time points, with an interaction between MWI and time. The worsening slope is due to the significant interaction between MWI and time

In model 2, we added an interaction term between MWI and time and adjusted for the same covariates as in Model 1. Adding this interaction term allowed us to assess whether the rates of progression in disability and functional decline according to multimorbidity and time. To illustrate the effect of significant interactions between MWI and time we calculated participant-specific predicted values for the disability outcome for an exemplary participant with the mean age and covariates (Fig. 1). We also modeled the association of worsening MWI (i.e., current multimorbidity plus incident conditions) over time, compared with constant MWI (i.e., current multimorbidity without additional incident conditions) over time, with the outcomes for the same exemplary participant.

Statistical significance was set at a 2-sided p-value < 0.05 for all analyses, which were conducted using SAS 9.4 (SAS Institute, Cary, NC) and Stata version 14.0 (StataCorp, College Station, TX, 2015).

## Results

### Participant characteristics

Baseline characteristics for the two analytic samples, 1) disability, and 2) gait speed and grip strength, are shown in Table 1. For the primary cohort, disability, the mean age was 67.3 (SD 9.7) years, and 57.8% were women. Most participants were White, married or living with a partner, and overweight. The MWI ranged from 0.0 to 30.5 (median 0.4), the number of ADL limitations ranged from 0 to 6 (median 0), and the number of IADL limitations from 0 to 5 (median 0). MWI increased over time (Supplemental Fig. 2).

**Table 1:** Baseline characteristics of the participants in the samples used for the disability analysis and in the sample used for the gait speed and grip strength analysis

**Characteristic**	**Disability** (*N* = 16,616)	**Gait speed & grip strength** (*N* = 2,748)
Age, years	67.3 (9.7)	72.8 (6.0)^a^
Sex, female, N (%)	9,610 (57.8)	1,545 (56.2)
*Ethnicity, N (%)*		
White	12,789 (77.0)	2,257 (82.1)
Black	2,232 (13.4)	281 (10.2)
Hispanic	1,272 (7.7)	168 (6.1)
Other	323 (1.9)	42 (1.5)
*Marital status, N (%)*		
Married/living with partner	10,762 (64.8)	13,629 (65.9)
Unmarried, living alone	3,705 (22.3)	636 (23.1)
Unmarried, living with other	2,149 (12.9)	286 (10.4)
*Education, N (%)*		
Less than high school	4,698 (28.3)	555 (20.2)
High school	5,696 (34.3)	1,021 (37.2)
College	3,118 (18.8)	558 (20.3)
≥ 4 years of college	3,104 (18.7)	614 (22.3)
*Household net worth, N (%)*^*b*^		
Quartile 1	4,153 (25.0)	687 (25.0)
Quartile 2	4,173 (25.1)	689 (25.1)
Quartile 3	4,135 (24.9)	687 (25.0)
Quartile 4	4,155 (25.0)	685 (24.9)
*Smoking status, N (%)*		
Never smoker	6,920 (41.7)	1,253 (45.6)
Past smoker	7,263 (43.7)	1,277 (46.5)
Current smoker	2,433 (14.6)	218 (7.9)
*Multimorbidity-Weighted Index*		
Mean (SD)	2.9 (3.8)	6.0 (5.3)
Median (IQR)	0.4 (0.0, 4.7)	4.6 (0.8, 8.6)
*Number of ADL limitations*		
Mean (SD)	0.4 (1.1)	0.2 (0.7)
Median (IQR)	0.0 (0.0, 0.0)	0.0 (0.0, 0.0)
*Number of IADL limitations*		
Mean (SD)	0.3 (0.8)	0.1 (0.4)
Median (IQR)	0.0 (0.0, 0.0)	0.0 (0.0, 0.0)
*Number of ADL* + *IADL limitations*		
Mean (SD)	0.7 (1.7)	0.3 (0.9)
Median (IQR)	0.0 (0.0, 0.0)	0.0 (0.0, 0.0)
Gait speed, m/s	-^c^	0.8 (0.2)
Grip strength, kg	-^c^	31.2 (10.6)

Abbreviations: *ADLs* Activities of Daily Living, *HRS* Health and Retirement Study, *IADLs* Instrumental Activities of Daily Living, *IQR* Interquartile range, *N* Number, *SD* Standard deviation

^**a**^Gait speed was only assessed in participants aged 65 years or older, so an older mean age is expected compared with the full HRS sample

^**b**^Household net worth quartiles for disability sample: Quartile 1: < $40,850, Quartile 2: $40,850-$122,000, Quartile 3: $122,001-$298,771, Quartile 4: > $298,771. For gait speed & grip strength sample: Quartile 1: ≤ $96,000, Quartile 2: $96,001-$243,000, Quartile 3: $243,001-$541,000, Quartile 4: > $541,000

^**c**^This measure was not available at baseline (2000) for the disability cohort, since those measurements started only in 2006

**Fig. 2:**
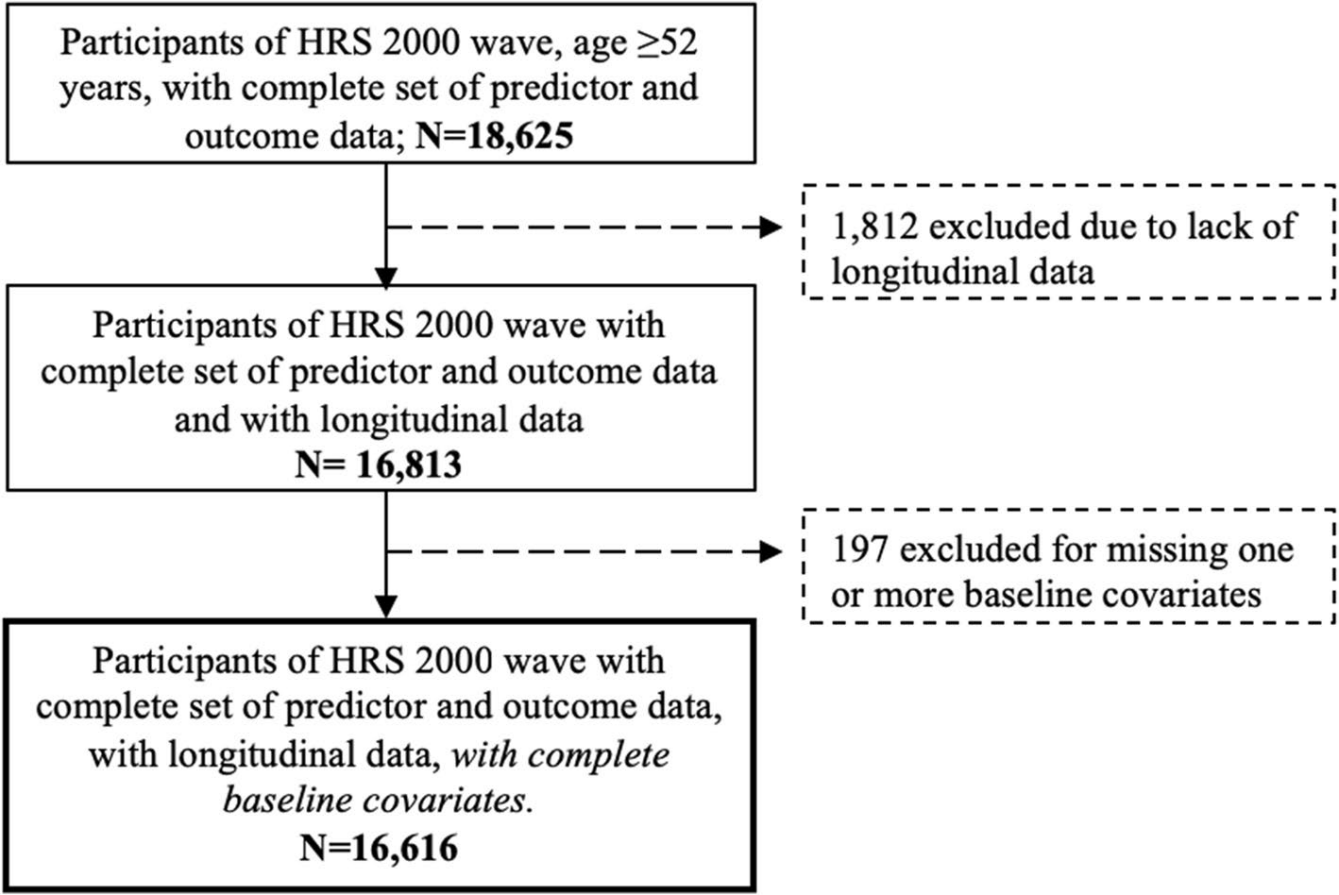
Consort diagram for the disability sample

### Multimorbidity and disability

Among 18,625 participants in the 2000 HRS wave with predictor and outcome data, we excluded 1,812 who lacked a follow-up measure of either the predictor or outcome and 197 who were missing baseline covariate data. The resulting analytical sample included 16,616 participants with longitudinal follow-up data ranging from one to eight follow-up observation(s) for the disability outcome analysis (Fig. 2). We observed different baseline characteristics between included and excluded participants (Supplemental Tables S2 and S3). Excluded participants who were alive at the end of follow-up (*N* = 387/2,009) were younger and healthier (lower MWI) on average, while excluded participants (*N* = 1,622/2,009) who died during follow-up were older and sicker (higher MWI) on average. ADL/IADL limitations and gait speed/grip strength at the end of follow-up for the two cohorts are described in Supplemental Tables S4 and S5.

Each additional one-point increase in MWI was associated with a 10% increase in incidence rate ratio of disability (IRR 1.10, 95% CI: 1.09, 1.10; Table 2). The incidence rate ratio of disability increased by 13% each year (IRR 1.13, 95%CI: 1.12 to 1.14), with a significant interaction between MWI and time (*p *< 0.0001; Table 2, Fig. 1).

**Table 2:** Adjusted changes and 95% CIs in predicted functional limitation, gait speed, and grip strength with multimorbidity for each 1 point-increase in the Multimorbidity-Weighted Index

	**Disability (*N* = 16,616)**		**Gait speed (*N* = 2,748)**		**Grip strength (*N* = 2,748)**	
	**Model 1** IRR (95% CI)	**Model 2** IRR (95% CI)	**Model 1** β (95% CI)	**Model 2** β (95% CI)	**Model 1** β (95% CI)	**Model 2** β (95% CI)
**MWI**	**1.07** **(1.07, 1.08)** ***p* < 0.0001**	**1.10** **(1.09, 1.10)** ***p* < 0.0001**	**-0.004** **(-0.005, -0.003)** ***p* < 0.0001**	**-0.004** **(-0.006, -0.001)** ***p* < 0.0001**	**-0.06** **(-0.09, -0.04)** ***p* < 0.0001**	**-0.01** **(-0.73, 0.04)** ***p* < 0.0001**
**Time**	**1.10** **(1.09, 1.10)** ***p* < 0.0001**	**1.13** **(1.12, 1.14)** ***p* < 0.0001**	**-0.01** **(-0.02, -0.01)** ***p* < 0. 0001**	**-0.01** **(-0.02, -0.01)** ***p* = 0.004**	**-0.66** **(-0.72, -0.60)** ***p* < 0.0001**	-0.59 ( -0.68, -0.49) *p* = 0.63
MWI*Time	-	**0.998** **(0.997, 0.998)** ***p* < 0.0001**	-	0.00002 (-0.0004, 0.0004) *p* = 0.92	-	-0.008 (-0.02, 0.0005) *p *= 0.06

Abbreviations: *β* Regression coefficient, *IRR* Incidence rate ratio, *MWI* Multimorbidity-Weighted Index

Legend:

Significant results are bolded

Covariates: sex, ethnicity (Non-Hispanic White, Non-Hispanic Black, Hispanic, Other), years of education (less than high school, high school, college, ≥ 4 years of college), BMI category (< 18.5, 18.5–24.9, 25–29.9, ≥ 30 kg/m^2^), smoking status (never smoked, past smoker, current smoker), living arrangement (married, unmarried and lives with another, unmarried and lives alone), household net worth by quartiles, time (years since baseline)

Model 1 (modeling change in intercept): independent variables included the predictor time-varying MWI, all covariates, and outcome measure at baseline for each respective outcome (i.e., disability at baseline for disability model, gait speed at baseline for gait speed model or grip strength at baseline for grip strength model)

Model 2 (modeling change in slope): Model 1 with additional interaction between MWI and time

### Multimorbidity and decline in physical functioning

Among 3,976 HRS participants in the 2006 HRS wave (baseline for the gait speed and grip strength analyses) with predictor and outcome data, 1,202 had no follow-up measures of either predictor or outcome and 26 were missing baseline covariate data, resulting in an analytical sample of 2,748 for the gait speed and grip strength analyses (Supplementary Figure S1). Each participant had longitudinal follow-up data ranging from one to two follow-up observations.

**Fig. 3:**
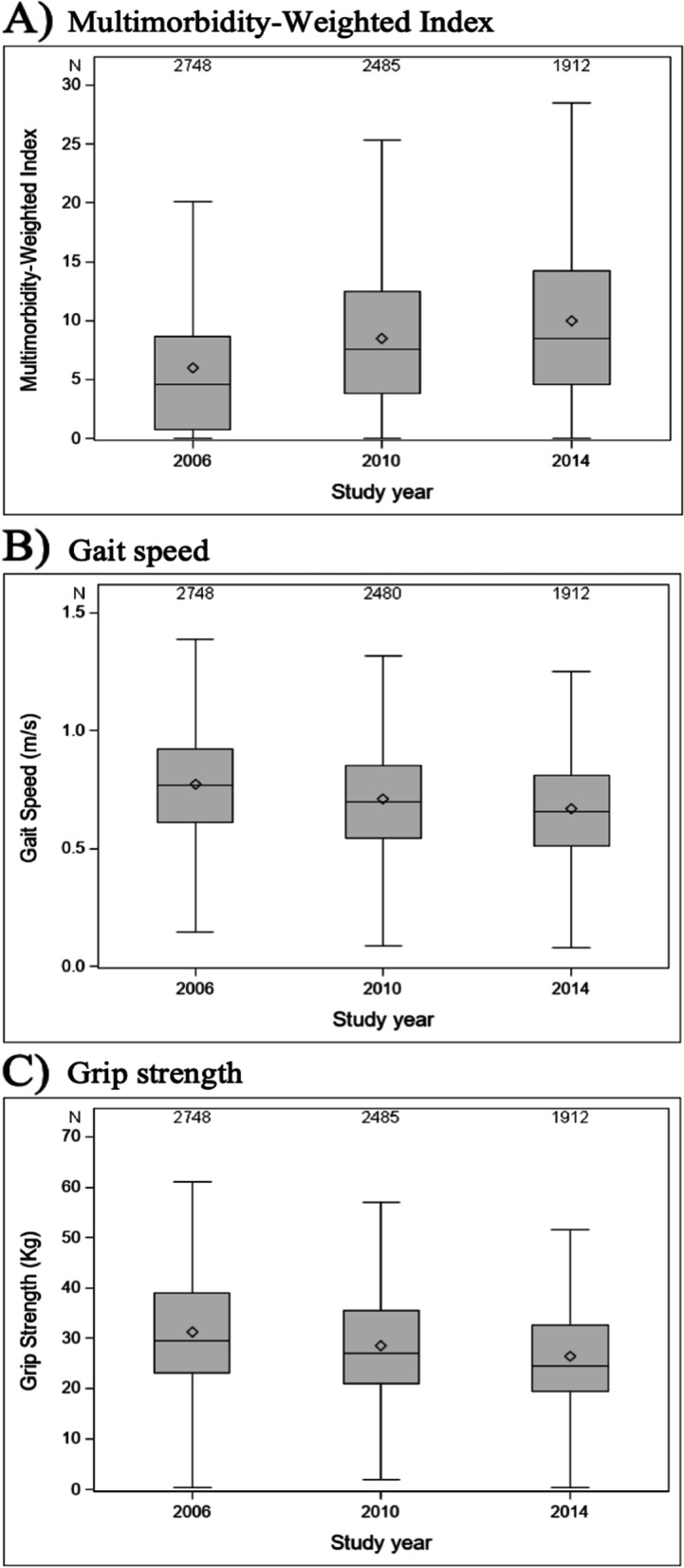
Box and whisker plots depicting the distributions of **A**) Multimorbidity-Weighted Index, **b**) gait speed, and **C**) grip strength at the three available time points in the gait speed and grip strength sample. The diamond inside the box represents the mean. The horizontal line inside the box represents the median. The box represents the interquartile range. The vertical lines extending from the box represent the minimum and maximum values. Outliers are not shown in the graph but were included in the analysis

Each additional one-point increase in MWI was associated with a decrease of 0.004 m/s (95% CI: -0.006, -0.001) in gait speed. The association between MWI and grip strength was not significant (regression coefficient 0.01, 95% CI: -0.07, 0.04; Table 2, Fig. 3). Each year, gait speed decreased by 0.01 m/s (95%CI: -0.02, -0.01), and grip strength by 0.59 kg (95%CI: -0.68, -0.49). We did not observe a significant interaction between MWI and time (*p*-value for interaction for gait speed 0.92, and for grip strength 0.06; Table 2).

## Discussion

In this large cohort representative of US adults aged 52 years and older, we found that multimorbidity, with conditions weighted to current physical functioning using the MWI, was associated with long-term disability and objective decline in gait speed. Further, the rate of disability progression accelerated as multimorbidity increased with time. The MWI is a tool that can be used to identify adults at higher risk for early and accelerated functional limitations who may benefit from preventive interventions to delay the onset and progression of disability. Potential implications include reducing downstream sequelae such as institutionalization, mortality, and loss of functional independence, a vital person-centered outcome [16, 35, 36].

The association between multimorbidity and long-term disability and decline in gait speed observed in this cohort corroborates and builds upon findings of most prior studies [11, 24, 37–43]. For example, Rigler et al. reported an odds of 2.30 (95%CI 1.09, 5.09) for developing a new ADL limitation over one year among community-dwelling older adults with chronic conditions in two different organ systems and an odds of 2.96 (95%CI 1.48, 6.25) for those with chronic conditions in three or more organ systems after adjusting for baseline functional status [43]. However, limitations in previous cohort studies include a shorter follow-up, use of a simple disease count (rather than a more comprehensive, robust, and validated measure of multimorbidity such as the MWI), not weighting multimorbidity with person-centered outcomes such as functioning, assessment of functioning using only subjective measures (e.g., ADLs, IADLs, SF-36, EQ-5D), and examining multimorbidity as a static exposure rather than accounting for the time-varying evolution of multimorbidity over prolonged follow-up. The different measures of multimorbidity and functioning used in prior studies limit our ability to compare effect sizes in prior studies and in our cohort.

In prior studies of direct head-to-head comparisons between MWI with simple disease count and mortality-based comorbidity measures such as the Charlson Comorbidity Index, [44] the MWI had the best model fit for predicting long-term physical functioning, although these studies did not examine the current outcomes of disability, grip strength and gait speed [21, 24]. While two cohort studies reported no significant association between multimorbidity and functional decline over time, they were limited to older adults (aged ≥ 65 [45] or ≥ 70 years [46]) who were mostly independent or prefrail at baseline. In our large cohort that included younger middle-aged participants, the prevalence of disability and poor physical functioning was highly skewed and overall low at baseline. It is possible that our sample population was healthier on average, but after even longer follow-up and higher accrual of multimorbidity, we might observe larger or faster rates of physical functioning decline and disability.

Our study illustrates the association of multimorbidity with disability and an objective measurement of physical functioning (i.e., gait speed) as multimorbidity progresses over time. Among participants with higher multimorbidity, we observed a faster rate of disability progression and decline in gait speed. We depicted hypothetical trajectories of multimorbidity over 16 years of follow-up in Fig. 1. These results demonstrate the importance of accounting for multimorbidity progression over time rather than treating multimorbidity as a static exposure at baseline.

Although we observed a statistically significant association between multimorbidity and decline in gait speed, the magnitude change for each additional point increase in MWI was small. To place our results in clinical context, a pooled analysis of nine cohort studies including individual data from 34,485 older adults followed for six to 21 years reported a 12% reduction in survival per 0.1 m/s decrease in gait speed [47]. While the magnitude of our association between MWI and gait speed was less than that associated with increased mortality risk, it is important to note that the 0.004 m/s decrease is for each one-point increase in MWI, so this could become clinically relevant as MWI worsens over time.

We assessed whether multimorbidity was associated with future physical functioning decline, but it is also possible that disability and physical functioning impairment worsen multimorbidity. For example, patients with difficulty managing their medications or transporting to medical appointments may experience disease exacerbation and progression due to suboptimal management of their chronic conditions. Furthermore, they may be unable to engage in physical activity and participate in social activities, [48] which are important for physical and mental health. Thus, a bidirectional association of multimorbidity with disability or physical functioning impairment may further negatively impact other downstream health outcomes.

The exclusion of participants who were on average older with a higher MWI (more chronic conditions and worse physical functioning) would likely result in an underestimation of our results, presuming that older and sicker adults would experience disability and objective functional decline prior to mortality. Thus, the true impact of MWI on future disability and objective physical functioning decline is likely even greater than reported in our results.

This study has important implications. Disability and impaired physical functioning represent a significant burden for individual physical and mental well-being, and healthcare systems and society, as the prevalence of adults with multimorbidity and disability increase. There is some evidence from randomized controlled trials that interventions by occupational and physical therapists can improve functioning, and potentially even reduce mortality over time [49, 50]. It is therefore important that healthcare providers identify individuals at risk for developing disability and physical functioning decline, in order to refer them early for preventive interventions. The MWI appears to be a systematic and efficient tool to help in this identification. However, interventions that prioritize the preservation of functioning in adults with multimorbidity remain highly needed [11].

### Strengths and limitations

This study has several limitations. First, the chronic conditions used to compute the MWI were self-reported. Although this carries the risk of under- or overreporting, administrative data can also lack complete diagnosis documentation, or contain errors or miscoding. Furthermore, self-reporting provides a patient view of diseases, which may be of particular relevance. Second, the HRS assessed only 15 of the 74 chronic conditions weighted in the MWI. However, the MWI has been externally validated in the HRS with 15 conditions [21]. Further, those 15 conditions were prevalent and highly associated with functioning in the original development study of the MWI, affected several body systems, and represent the most frequently assessed conditions in other surveys and those used in multimorbidity measures. Third, only five IADLs were assessed in the HRS, so disability may have been underestimated. If the true number of disability limitations were higher, it is possible that a larger magnitude or rate of worsening disability may have been observed. Finally, the HRS included only adults aged 52 years or older, and gait speed was assessed only in respondents aged 65 years and older, so we cannot generalize our findings to younger adults. Nevertheless, disability, physical functioning impairment and multimorbidity are more prevalent and progress more in older adults, so data in older adults is of particular clinical relevance.

Our study has several strengths. First, we assessed a very large sample of adults representative of the US population, with a longer follow-up period than previous studies [11]. Second, we assessed both subjective and objective measures of physical functioning and disability with repeated measures spanning over a decade. Third, we assessed the interaction between multimorbidity and time to assess the rates of decline in physical functioning and disability over 8–16 years, illustrated by distinct participant trajectories based on our models. Finally, we assessed multimorbidity by weighting the chronic conditions based on current functioning, which is a person-centered outcome that also provides a better model fit than does simple disease count and mortality-weighted measures when studying the association with disability and decline in physical functioning [21, 24].

## Conclusion

In conclusion, in this analysis of a large nationally-representative US cohort of adults aged 52 years and older, multimorbidity, as weighted to physical functioning, was associated with worse disability, including faster rates of disability progression, and a decrease in an objective measure of physical functioning over a 16-year follow-up. The MWI may serve as a useful tool for early identification of adults at highest risk for disability and physical functioning decline. This can help to better target preventive interventions to efficiently reduce the sequalae of physical impairment and extend disability-free survival.

## Supplementary Information

**Additional file 1:** **Supplemental Table S1.** Weights for the calculation of the MWI using all 15 self-reported physician-diagnosed chronic conditions available in the HRS.* Supplemental Table S2. Comparison of included and all (i.e., both alive and dead at the end of follow-up) excluded participants. **Supplemental Table S3.** Comparison of included participants and excluded participants who were alive at end of follow-up. **Supplemental Table S4.** ADL/IADL limitations at the end of follow-up in the disability cohort. **Supplemental Table S5.** Gait speed and grip strength at the end of follow-up in gait speed/grip strength cohort. **Supplemental Figure S1. **Consort diagram for the gait speed and grip strength sample. **Supplemental Figure S2**. Box and whisker plots depicting MWI over the 16-year time period in the disability sample (*N*=16,616). The diamond inside the box represents the mean. The horizontal line inside the box represents the median. The box represents the interquartile range. The vertical lines extending from the box represent the minimum and maximum values. Outliers are not shown in the graph (for compliance with HRS data reporting) but were included in the analysis.

## Data Availability

HRS data are publicly available. Raw data and statistical coding used for the current study are available by the senior author upon request at the following e-mail address: MYWei@mednet.ucla.edu.

## Abbreviations

ADLs: Activities of Daily Living
BMI: Body mass index
CI: Confidence interval
HRS: Health and Retirement Study
IADLs: Instrumental Activities of Daily Living
IRR: Incidental Rate Ratio
MWI: Multimorbidity-Weighted Index
SD: Standard Deviation
