# Supplementary material for: Multimorbidity and long-term disability and physical functioning decline in middle-aged and older Americans: an observational study
Source: BMC Geriatr. 2022 Nov 28;22:910. doi: 10.1186/s12877-022-03548-9 (PMC9703785; doi:10.1186/s12877-022-03548-9)

**SUPPLEMENTARY INFORMATION**

**Multimorbidity and long-term disability and physical functioning decline**

**in middle-aged and older Americans**

Carole E. Aubert, Mohammed U. Kabeto, Navasuja Kumar, Melissa Y. Wei

**Supplemental Table S1.** Weights for the calculation of the MWI using all 15 self-reported physician-diagnosed chronic conditions available in the HRS.*

| **Chronic condition** | **Weight** |
| --- | --- |
| Dementia | 6.10 |
| Lung disease | 4.32 |
| Arthritis | 3.52 |
| Congestive heart failure | 4.76 |
| Stroke | 3.79 |
| Knee replacement | 9.11 |
| Diabetes | 2.67 |
| Connective tissue disease | 3.02 |
| Arrhythmia | 1.33 |
| Angina | 2.20 |
| Hypertension | 0.343 |
| Glaucoma | 0.427 |
| Hip replacement | 3.55 |
| Myocardial infarction | 1.73 |
| Other heart condition | 0.90 |
| Cancer, except skin cancer | 0.83 |
| **Maximum possible** | **47.77** |

**Legend:** In the HRS, 15 chronic conditions were available. The MWI is calculated by adding the weights of each present condition. For example, if a person has dementia, stroke and diabetes, then MWI = 6.10 + 3.79 + 2.67 = 12.56. With these 15 conditions, the MWI ranges from 0 (in persons with none of the 15 conditions) to 47.77 (in persons with all 15 conditions).

*based on Wei et al. Multimorbidity and Physical and Cognitive Function: Performance of a New Multimorbidity-Weighted Index. The journals of gerontology Series A, Biological sciences and medical sciences 2018;73:225-32.

**Supplemental Table S2.** Comparison of included and all (i.e., both alive and dead at the end of follow-up) excluded participants.

| Variable | Included in the analyses (N=16,616) | Excluded from the analyses (N=2,009)* | p-value** |
| --- | --- | --- | --- |
| **Age**  Mean (SD)  Median (IQR) | 67.3 (9.7)  66.0 (60.0, 74.0) | 74.0 (11.3)  75.0 (65.0, 83.0) | <0.0001 |
| **Sex, N (%)** | | | <0.0001 |
| Male | 7,006 (42.2) | 939 (46.7) |  |
| Female | 9,610 (57.8) | 1,070 (53.3) |  |
| **Education, N (%)** | | | <0.0001 |
| Less than high school | 4,698 (28.3) | 819 (40.8) |  |
| High school | 5,696 (34.3) | 579 (28.8) |  |
| College | 3,118 (18.8) | 338 (16.8) |  |
| ≥4 years of college | 3,104 (18.7) | 273 (13.6) |  |
| **Multimorbidity-weighted index**  Mean (SD)  Median (IQR) | 2.9 (3.8)  0.4 (0.0, 4.7) | 5.5 (5.2)  4.6 (0.3, 8.7) | <0.0001 |
| **Number of ADL + IADL limitations**  Mean (SD)  Median (IQR) | 0.7 (1.7)  0.0 (0.0, 0.0) | 2.5 (3.5)  1.0 (0.0, 4.0) | <0.0001 |
| **Number of ADL limitaitons**  Mean (SD)  Median (IQR) | 0.4 (1.1)  0.0 (0.0, 0.0) | 1.4 (2.0)  0.0 (0.0, 2.0) | <0.0001 |
| **Number of IADL limitations**  Mean (SD)  Median (IQR) | 0.3 (0.8)  0.0 (0.0, 0.0) | - 1. (1.7)   0.0 (0.0, 2.0) | <0.0001 |

**Abbreviations:** ADL, activities of daily living; IADL, instrumental activities of daily living; IQR, interquartile range; N, number; SD, standard deviation

***** Individuals not included in the analyses due to lack of longitudinal data (N=1,812) or baseline covariates (N=197).

** Chi-square test was used for categorical variables and Kruskal-Wallis test used for continuous variables.

**Supplemental Table S3.** Comparison of included participants and excluded participants who were alive at end of follow-up.

| Variable | Included in the analysis (N=16,616) | Excluded from the analyses and alive (N=387)* | p-value** |
| --- | --- | --- | --- |
| **Age**  Mean (SD)  Median (IQR) | 67.3 (9.7)  66.0 (60.0, 74.0) | 63.6 (7.9)  62.0 (58.0, 69.0) | <0.0001 |
| **Sex, N (%)** |  |  | 0.67 |
| Male | 7,006 (42.2) | 159 (41.1) |  |
| Female | 9,610 (57.8) | 228 (58.9) |  |
| **Education n (%)** |  |  | 0.023 |
| Less than high school | 4,698 (28.3) | 91 (23.5) |  |
| High school | 5,696 (34.3) | 122 (31.5) |  |
| College | 3,118 (18.8) | 87 (22.5) |  |
| ≥4 years of college | 3,104 (18.7) | 87(22.5s) |  |
| **Multimorbidity-weighted index**  Mean (SD)  Median (IQR) | 2.9 (3.8)  0.4 (0.0, 4.7) | 1.7 (2.8)  0.3 (0.0, 2.7) | <0.0001 |
| **Number of ADL + IADL limitations**  Mean (SD)  Median (IQR) | 0.7 (1.7)  0.0 (0.0, 0.0) | 0.3 (1.1)  0.0 (0.0, 0.0) | <0.0001 |
| **Number of ADL limitations**  Mean (SD)  Median (IQR) | 0.4 (1.1)  0.0 (0.0, 0.0) | 0.2 (0.7)  0.0 (0.0, 0.0) | <0.0001 |
| **Number of IADL limitations**  Mean (SD)  Median (IQR) | - 1. (0.8)   0.0 (0.0, 0.0) | 0.1 (0.5)  0.0 (0.0, 0.0) | 0.0045 |

**Abbreviations:** ADL, activities of daily living; IADL, instrumental activities of daily living; IQR, interquartile range; N, number; SD, standard deviation

***** Among the 2,009 participants excluded from the analysis, 1,622 died before/during 2016. Among them, 1,308 (80.6%) died between 2000-2002, and were the unable to contribute to longitudinal data.

** Chi-square test was used for categorical variables and Kruskal-Wallis test used for continuous variables.

**Supplemental Table S4.** ADL/IADL limitations at the end of follow-up in the disability cohort.

| **Outcome at last visit** | **Disability cohort (N=16,616)** |
| --- | --- |
| **Number of ADL limitations**  Mean (SD)  Median (IQR) | 1.4 (2.0)  0.0 (0.0, 2.0) |
| **Number of IADL limitations**  Mean (SD)  Median (IQR) | 1.1 (1.7)  0.0 (0.0, 2.0) |
| **Number of ADL + IADL limitations**  Mean (SD)  Median (IQR) | 2.5 (3.5)  1.0 (0.0, 4.0) |

**Abbreviations:** ADL, activities of daily living; IADL, instrumental activities of daily living; IQR, interquartile range; N, number; SD, standard deviation

**Supplemental Table S5.** Gait speed and grip strength at the end of follow-up in gait speed/grip strength cohort.

| **Outcome at last visit** | **Gait speed/grip strength cohort (N=2,748)** |
| --- | --- |
| **Gait speed (m/s)**  Mean (SD)  Median (IQR) | 0.6 (0.2)  0.6 (0.5, 0.8) |
| **Grip strength (kg)**  Mean (SD)  Median (IQR) | 26.5 (9.5)  25.0 (19.5, 33.0) |

**Abbreviations:** ADL, activities of daily living; IADL, instrumental activities of daily living; IQR, interquartile range; N, number; SD, standard deviation

**Supplemental Figure S1.** Consort diagram for the gait speed and grip strength sample.


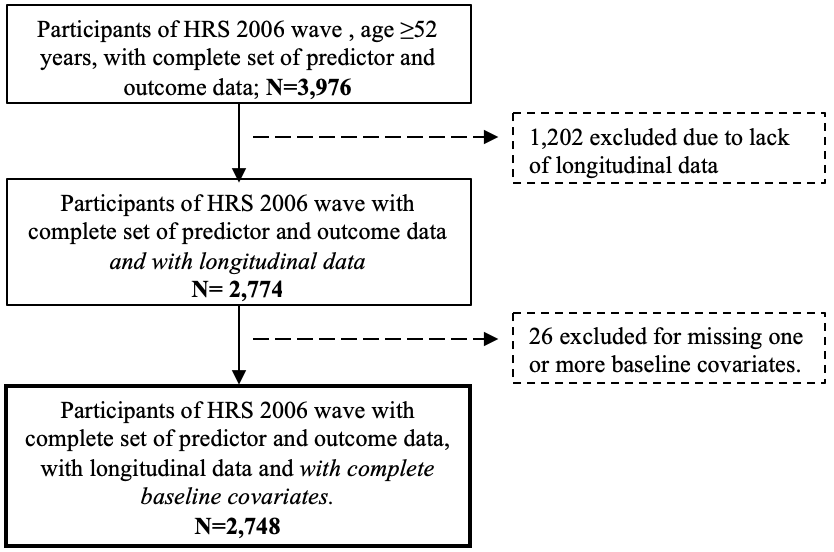


**Supplemental Figure S2**. Box and whisker plots depicting MWI over the 16-year time period in the disability sample (N=16,616). The diamond inside the box represents the mean. The horizontal line inside the box represents the median. The box represents the interquartile range. The vertical lines extending from the box represent the minimum and maximum values. Outliers are not shown in the graph (for compliance with HRS data reporting) but were included in the analysis.


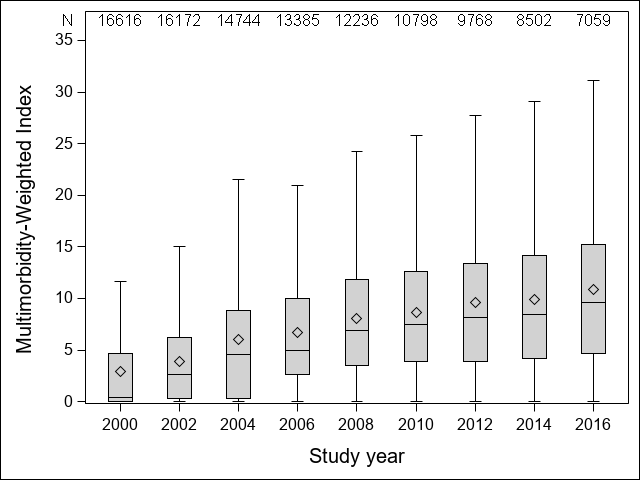

Supplement: Supplementary file 1 — Additional file 1: Supplemental Table S1. Weights for the calculation of the MWI using all 15 self-reported physician-diagnosed chronic conditions available in the HRS.* Supplemental Table S2. Comparison of included and all (i.e., both alive and dead at the end of follow-up) excluded participants. Supplemental Table S3. Comparison of included participants and excluded participants who were alive at end of follow-up. Supplemental Table S4. ADL/IADL limitations at the end of follow-up in the disability cohort. Supplemental Table S5. Gait speed and grip strength at the end of follow-up in gait speed/grip strength cohort. Supplemental Figure S1. Consort diagram for the gait speed and grip strength sample. Supplemental Figure S2. Box and whisker plots depicting MWI over the 16-year time period in the disability sample (N=16,616). The diamond inside the box represents the mean. The horizontal line inside the box represents the median. The box represents the interquartile range. The vertical lines extending from the box represent the minimum and maximum values. Outliers are not shown in the graph (for compliance with HRS data reporting) but were included in the analysis. [file 12877_2022_3548_MOESM1_ESM.docx]
